# Chondrocalcinosis is common in the absence of knee involvement

**DOI:** 10.1186/ar4043

**Published:** 2012-10-04

**Authors:** Abhishek Abhishek, Sally Doherty, Rose Maciewicz, Kenneth Muir, Weiya Zhang, Michael Doherty

**Affiliations:** 1 Academic Rheumatology, University of Nottingham, Nottingham NG5 1PB, UK; 2 Respiratory and Inflammation iMed, AstraZeneca, MöIndal, Sweden; 3 Health Sciences Research Institute, University of Warwick, Warwick CV4 7AL, UK

## Abstract

**Introduction:**

We aimed to describe the distribution of radiographic chondrocalcinosis (CC) and to examine whether metacarpophalangeal joint (MCPJ) calcification and CC at other joints occurs in the absence of knee involvement.

**Methods:**

This was a cross-sectional study embedded in the Genetics of Osteoarthritis and Lifestyle study (GOAL). All participants (*n *= 3,170) had radiographs of the knees, hands, and pelvis. These were scored for radiographic changes of osteoarthritis (OA), for CC at knees, hips, symphysis pubis, and wrists, and for MCPJ calcification. The prevalence of MCPJ calcification and CC overall, at each joint, and in the presence or absence of knee involvement, was calculated.

**Results:**

The knee was the commonest site of CC, followed by wrists, hips, and symphysis pubis. CC was more likely to be bilateral at knees and wrists but unilateral at hips. MCPJ calcification was usually bilateral, and less common than CC at knees, hips, wrists, and symphysis pubis. Unlike that previously reported, CC commonly occurred without any knee involvement; 44.4% of wrist CC, 45.9% of hip CC, 45.5% of symphysis pubis CC, and 31.3% of MCPJ calcification occurred in patients without knee CC. Those with meniscal or hyaline articular cartilage CC had comparable ages (*P *= 0.21), and neither preferentially associated with fibrocartilage CC at distant joints.

**Conclusions:**

CC visualized on a plain radiograph commonly occurs at other joints in the absence of radiographic knee CC. Therefore, knee radiographs alone are an insufficient screening test for CC. This has significant implications for clinical practice, for epidemiologic and genetic studies of CC, and for the definition of OA patients with coexistent crystal deposition.

## Introduction

Chondrocalcinosis (CC) predominantly results from calcium pyrophosphate (CPP) crystal deposition (CPPD) [1]. Although the knee is the commonest location of CC, it is unclear whether the wrist [1-4], or the symphysis pubis is the second most commonly affected joint [5-7]. Similarly, the frequency with which CC occurs at other joints in the absence of knee involvement is unclear. Most studies suggest that CC occurs uncommonly or does not occur in the absence of knee involvement [1,3,4,6-10]. For example, a study using a complete skeletal survey of 48 participants suggested that hand, knee, and pelvis radiographs together were sufficient to identify patients with CC at any joint [1]. It also suggested that 89% of cases with CC at any site could be identified by using knee radiographs, and that this figure increased to 98% when both knees and pelvis were radiographed [1]. This observation promoted a notion that knee radiographs alone may be a sufficient screening test to identify most patients with CC. However, a couple of relatively small studies suggested that one fourth to one third of all CC cases did not have any knee involvement [2,5].

Establishing whether CC commonly occurs in those without knee involvement is important, as it will facilitate the diagnosis of arthropathy associated with CPPD, and will allow accurate ascertainment of cases and controls for research. The overall aim of this study was to examine the distribution of CC by using knee, pelvis, and hand radiographs. The objectives were to calculate the prevalence of CC at each joint and of calcification in the synovium and capsule of metacarpophalangeal joints (MCPJs), which associates with CC [11]; to compare the prevalence of CC at other joints and the prevalence of MCPJ calcification in the presence and absence of knee CC; and to examine whether fibrocartilage CC at a distant joint preferentially occurs in those with meniscal or hyaline articular cartilage CC at the knee.

## Materials and methods

This cross-sectional study used data from the Genetics of Osteoarthritis and Lifestyle (GOAL) study. GOAL comprises 3,170 individuals: 1,007 with clinically severe hip OA, 1,042 with clinically severe knee OA, and 1,121 without knee or hip OA, recruited from 2002 to 2006. The study was approved by the Nottinghamshire Research Ethics Committee, UK.

The details of GOAL have been published elsewhere [12]. In brief, all participants completed a detailed questionnaire, were examined by a research metrologist, gave urine and blood samples, and underwent the following standardized radiographic assessments: posteroanterior weight-bearing semiflexed knee radiographs by using the SynaFlexer positioning frame (Synarc); skyline views of patellofemoral joints; supine pelvis radiographs for hips and symphysis pubis; and anteroposterior hand views, which included the wrists.

Radiographs were graded for CC at the knees, hips, wrists, and symphysis pubis, for MCPJ calcification, and for structural changes of OA by a senior research metrologist (SD). CC was present if linear or nummular calcification in fibro- or hyaline articular cartilage was found; synovial/capsular calcification (occasionally also CC) was also recorded at the metacarpophalangeal joints (MCPJs). Knee CC was scored for compartmental (lateral or medial tibiofemoral joint (TFJ) compartment), and cartilaginous location (hyaline or fibrocartilage).

### Statistical analysis

Mean (standard deviation (SD)) and *n *(%) were used to describe continuous and categoric variables. Analysis of variance (ANOVA) was used to compare continuous variables in different categories. The prevalence and 95% confidence interval (CI) of CC (including MCPJ calcification) was calculated at each joint region for any, unilateral, bilateral, and isolated CC (that is, involvement of target joint without involvement of any distant joint). The 95% CI was calculated as $p\pm 1.96\times \sqrt{\frac{p\left(1-p\right)}{n}}$, where *p *is the prevalence and *n *is the number of participants. The prevalence (95% CI) of CC and MCPJ calcification at other joints in the presence or absence of knee CC also was calculated. The prevalence of symphysis pubis CC, a marker of fibrocartilage calcification, was compared between those with isolated meniscal CC, isolated hyaline cartilage CC, and both meniscal and hyaline articular cartilage CC at the knee. Statistical analysis was done by using SPSS v14.

## Results

The mean (SD) age of participants was 66.6 (7.9) years, and 1,536 (48.5%) were women. Radiographic knee OA and hip OA were present in 1,602 (51.0%) and 1,168 (36.9%) participants. Information about CC and MCPJ calcification at all joints radiographed was available for 3,118 participants who were included in the analysis.

### Distribution of chondrocalcinosis

**Figure 1:**
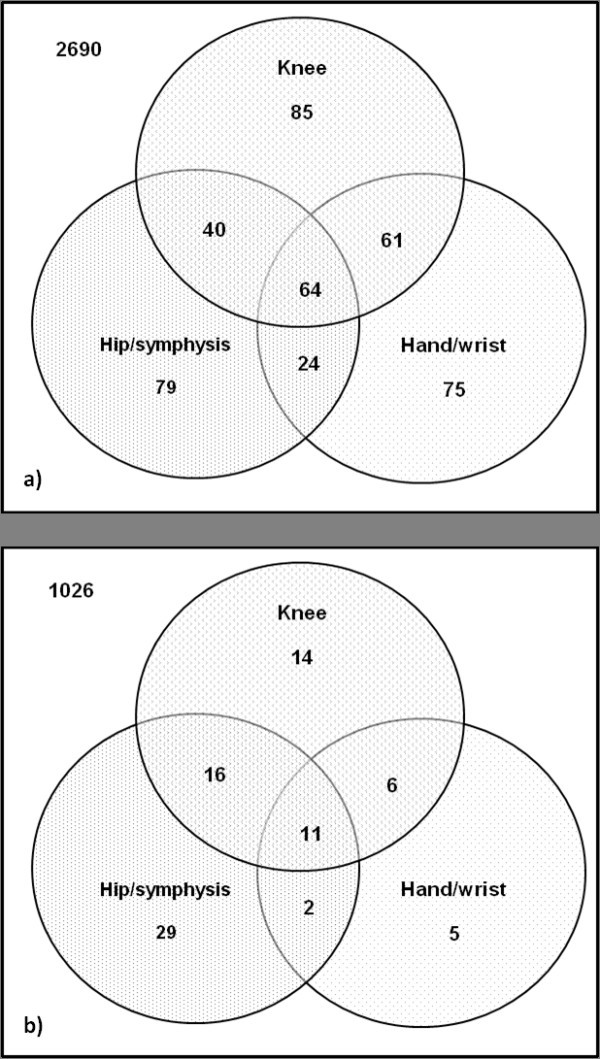
**Distribution of CC and metacarpophalangeal joint (MCPJ) calcification in knee, hand, and pelvis radiographs in (a) all participants (*n *= 3118), and in (b) participants without knee or hip OA (*n *= 1,109)**.

In total, 428 participants had MCPJ calcification or CC at any site (prevalence (95% CI) 13.7% (12.6% to 15.0%)). Knee was the commonest site involved (Table 1). The prevalence (95% CI) of CC at any knee, hip, or symphysis pubis; and of CC at any knee, wrist, or MCPJ calcification was 11.3% (10.3% to 12.4%), and 11.2% (10.1% to 12.3%), respectively. MCPJ calcification most commonly occurred at the index and middle fingers. CC at other joints and MCPJ calcification were common in the absence of knee involvement (Table 2). In participants with CC at any site or MCPJ calcification, only 58.4% (250 of 428), 52.3% (228 of 428), and 48.4% (207 of 428) could be detected by using knee, hand, and pelvis radiographs alone (Figure 1a). This increased to 82.5% (355 of 428), 81.5% (349 of 428), and 80.1% (343 of 428) when knee and pelvis, knee and hand, and hand and pelvis radiographs were examined in combination (Figure 1a).

**Table 1:** Prevalence of chondrocalcinosis (CC) and metacarpophalangeal joint (MCPJ) calcification

Index joint	Prevalence (95% CI) of CC					
	
	Overall (%)	Right (%)	Left (%)	Unilateral (%)	Bilateral (%)	Isolated (%)
Knee	8.0 (7.0-8.9)	6.7 (5.8-7.5)	6.0 (5.2-6.8)	3.3 (2.7-3.9)	4.7 (4.0-5.4)	2.7 (2.2-3.4)
Wrist	6.9 (6.0-7.8)	5.4 (4.6-6.2)	5.8 (5.0-6.6)	2.7 (2.1-3.2)	4.3 (3.6-4.9)	2.0 (1.6-2.6)
Hip	5.0 (4.3-5.9)	4.0 (3.3-4.7)	3.0 (2.4-3.6)	3.1 (2.5-3.7)	2.0 (1.5-2.5)	1.4 (1.0-1.9)
Symphysis	3.6 (2.9-4.2)	N/A	N/A	N/A	N/A	0.8 (0.5-1.2)
Any MCPJs	1.5 (1.1-2.0)	0.9 (0.6-1.2)	1.1 (0.7-1.4)	0.4 (0.2-0.6)	1.1 (0.8-1.5)	0.1 (0.0-0.3)

**Table 2:** Chondrocalcinosis (CC) at other sites and metacarpophalangeal joint (MCPJ) calcification in the presence or absence of knee involvement

Index joint	Knee CC +ve	Knee CC -ve	Proportion (95%CI) with CC at index joint without knee involvement
Wrist	120	96	44.4 (38.0-51.0)%
Hip	85	72	45.9 (38.3-53.4)%
Symphysis pubis	61	51	45.5 (36.6-54.8)%
Any MCPJs	33	15	31.3 (20.0-45.0)%

As GOAL participants have a high prevalence of hip or knee OA, and OA can affect the distribution of CC, data about distribution of CC were analyzed for 1,109 GOAL participants without radiographic hip or knee OA for whom CC could be scored at all joints examined. Eighty-three participants had MCPJ calcification or CC at any site. In this subset, only 56.7% (47 of 83), 28.9% (24 of 83), and 65.1% (58 of 83) participants with CC at any joint or MCPJ calcification could be detected on knee, hand, and pelvis radiographs alone (Figure 1b).

### Knee chondrocalcinosis

Knee CC was more common in the lateral TFJ compartment than in the medial TFJ compartment (right: 90.9% lateral, 76.1% medial; left: 87.2% lateral, 71.8% medial; *P *< 0.001). It affected the fibrocartilaginous meniscus more frequently than the intraarticular hyaline cartilage (right: 91.4% fibro-, 56.8% hyaline; left: 86.3% fibro-, 53.7% hyaline; *P *< 0.001). Similarly, isolated fibrocartilage involvement was more common than isolated hyaline cartilage involvement (right knee: 42.8% fibro-, 8.2% hyaline; left knee: 46.3% fibro-, 13.7% hyaline; *P *< 0.001). At the knee, patients with isolated meniscal CC, isolated hyaline articular cartilage CC, and CC in both fibro- and hyaline articular cartilage were of comparable age, with a mean (SD) age of 69.7 (6.7), 69.5 (7.4), and 71.0 (5.4) years, respectively (*P *= 0.21).

### Symphysis pubis chondrocalcinosis

Of the 3,131 participants for whom information about knee and symphysis pubis CC was available, isolated hyaline cartilage CC, isolated meniscal CC, and both meniscal and hyaline cartilage CC at the knee were present in 20, 97, and 130 participants, respectively; and the prevalence (95% CI) of symphysis pubis CC in these groups was 10% (2.8% to 30.0%), 25.8% (18.1% to 35.3%), and 26.2% (19.4% to 34.3%), respectively. Knee CC was absent in 2,884 participants, and 1.8% (1.4% to 2.3%) of these had symphysis pubis CC.

## Discussion

This is the first large study to examine the distribution of CC and MCPJ calcification by using knee, hand, and pelvis radiographs [1,5,6]. We report that hip, wrist, and symphysis pubis CC and MCPJ calcification commonly occur without knee involvement. We also found that wrist CC is more common than hip CC, and that CC is more likely to be bilateral, except at the hips. We confirmed previous observations that the lateral TFJ compartment [5,9,13,14] and the menisci are the preferred sites for knee CC [2,5,7,8,11].

In our study, 42% of the CC cases had no knee involvement. This suggests that a significant proportion of cases with CC would be missed if knee radiographs alone were used to screen for CC, and this would result in important misclassification (for example, in genetic case-control studies). Although similar findings have been reported in two small studies [2,5], this is discordant with most previous reports [1,3,4,6-10,15]. Previous studies were small, hospital based, and were therefore likely to overrepresent symptomatic knee arthropathy cases. Similarly, in our study, only 80% to 82% of cases with CC at any site or MCPJ calcification could be identified by using radiographs of any two regions alone. This suggests that radiographs of knees, hips, and hands should be performed to screen adequately for CC, and is not in keeping with a previous study that suggested that 98% of cases with CC could be identified by using pelvis and knee radiographs alone [1].

Despite a high prevalence of OA, the prevalence of CC at knees, knees and pelvis, and knees and wrists reported here are similar to those in community studies [9,14,15]. GOAL participants have a high prevalence of large-joint OA and would be expected to have a twofold greater prevalence of CC [14]. However, they were also approximately 10 years younger than those in population studies of CC [9,14,15]. Because the prevalence of CC halves for each 10-year decrease in age, this probably compensated for any increase in prevalence of CC resulting from a high prevalence of OA [14].

Because this is the largest study to compare the prevalence of CC at wrists and hips, we can infer that the wrist is the second most common site for CC. In our study, CC was more common at the hips than at the symphysis pubis [5-7]. However, in one large community-based survey, symphysis pubis calcification was 10-fold more common than hip CC [9]. The difference in prevalence between this survey and our study may again result from the high prevalence of severe symptomatic hip or knee OA and the younger age of GOAL participants, which might increase the prevalence of CC at the hips but not at the symphysis pubis.

As reported previously, no predilection was found for CC to occur on any side [14]. However, a study reported unilateral CC preferentially affecting the right knee [9]. In agreement with other reports, CC was more likely to be bilateral at the knees and wrist [2-5,8,9,11,14]. However, unlike prior studies, CC was likely to be unilateral at the hips [2,4,5,8,11]. More than 90% of cases with MCPJ calcification had CC at other joints, suggesting that MCPJ calcification is part of CPPD. Similar findings have been reported before [11].

Patients with isolated meniscal CC, isolated hyaline cartilage CC, and CC in both meniscus and hyaline cartilage of the knee were of similar age. This suggests that knee CC occurs at the same time in both fibro- and hyaline articular cartilage. However, this should be confirmed in a community-based survey to exclude any effect of healthcare seeking bias. Moreover, fibrocartilage CC at a distant joint (for example, symphysis pubis) was not significantly more common in those with isolated meniscal CC, or CC at both meniscus and hyaline articular cartilage than in those with isolated hyaline cartilage CC. We were unable to examine whether hyaline cartilage CC at the hip is more common in those with hyaline cartilage CC at the knee, because confident specification of tissue localization of CC at the hip is difficult by using a single-view plain radiograph. Further macroradiographic studies are warranted to examine the prevalence of fibro- and hyaline articular cartilage CC at the hip, and to examine their relation with fibro- and hyaline articular cartilage CC at the knee.

This is the largest study to examine the distribution of CC and MCPJ calcification. Several caveats to these findings exist. First, GOAL was designed as a case-control cohort to examine risk factors for large-joint OA. In the present study, we undertook a nested study that involved reattribution of case and control status. Two thirds of participants had severe symptomatic hip or knee OA, and the remaining have no radiographic or clinical features of hip or knee OA. The population under study therefore limits the generalizability of the findings. However, the prevalence of CC at the knee, knee and hip/symphysis pubis, knee and wrist reported in this study are similar to those observed in large community-based radiographic surveys [9,14,15], providing face validity. Moreover, a similar pattern was observed when data for patients without large-joint OA were analyzed. Finally, it is possible that knee CC detectable on ultrasound may be present in some participants who do not have visible CC on knee radiographs and have CC at other joints. However, plain radiographs are still the first-line radiographic examination in patients with arthropathy, and in routine clinical practice, not all patients undergo musculoskeletal ultrasound. The results of our study are therefore transferable to routine clinical practice, and to large epidemiologic studies of CC.

## Conclusions

CC at other joints and MCPJ calcification are common in the absence of knee involvement, and do not prefer one side over the other. In those with CC, wrist is the second most commonly involved site after the knee. Knee CC, wrist CC, and MCPJ calcification, but not hip CC, are more often bilateral. At the knee, CC is more common at the lateral TFJ compartment than in the medial TFJ compartment; and in the menisci than in hyaline cartilage. There was no evidence to suggest that either meniscal or hyaline articular cartilage CC precedes the other, or preferentially associates with fibrocartilage CC at distant joints. These findings derived from a hospital-based population should be confirmed in a general population-based cross-sectional study.

## Abbreviations

ANOVA: analysis of variance; CC: chondrocalcinosis; CI: confidence interval; CPP: calcium pyrophosphate crystal; CPPD: calcium pyrophosphate crystal deposition; GOAL: genetics of osteoarthritis and lifestyle; MCPJ: metacarpophalangeal joint; *n*: number of participants; OA: osteoarthritis; p: prevalence; SD: standard deviation; TFJ: tibiofemoral joint.

## Competing interests

AstraZeneca had no role in the study design, data analysis, and in drafting of this manuscript. Dr. Rose Maciewicz is an employee of AstraZeneca, owns shares in that company, and was involved as a scientific collaborator in the GOAL study. Other authors have no competing interests to declare.

## Authors' contributions

AA was involved in study conception and design and in analysis and interpretation of the data. SD was involved in study conception, data acquisition, and radiographic scoring. RM, KM, WZ, and MD were involved in study conception and design and in analysis and interpretation of the data. KM and MD were involved in data acquisition as well. All authors were involved in drafting the article or revising it, and all authors approved the final version.
